# Age-related hearing loss pertaining to potassium ion channels in the cochlea and auditory pathway

**DOI:** 10.1007/s00424-020-02496-w

**Published:** 2020-12-17

**Authors:** Barbara Peixoto Pinheiro, Barbara Vona, Hubert Löwenheim, Lukas Rüttiger, Marlies Knipper, Youssef Adel

**Affiliations:** 1 Translational Hearing Research, Tübingen Hearing Research Center, Department of Otolaryngology, Head and Neck Surgery, University of Tübingen, 72076 Tübingen, Germany; 2 Molecular Physiology of Hearing, Tübingen Hearing Research Center, Department of Otolaryngology, Head and Neck Surgery, University of Tübingen, 72076 Tübingen, Germany

**Keywords:** Kv7.4, Kv7.1, BK, SK2, Kv3.1, Presbycusis

## Abstract

Age-related hearing loss (ARHL) is the most prevalent sensory deficit in the elderly and constitutes the third highest risk factor for dementia. Lifetime noise exposure, genetic predispositions for degeneration, and metabolic stress are assumed to be the major causes of ARHL. Both noise-induced and hereditary progressive hearing have been linked to decreased cell surface expression and impaired conductance of the potassium ion channel K_V_7.4 (KCNQ4) in outer hair cells, inspiring future therapies to maintain or prevent the decline of potassium ion channel surface expression to reduce ARHL. In concert with K_V_7.4 in outer hair cells, K_V_7.1 (KCNQ1) in the stria vascularis, calcium-activated potassium channels BK (KCNMA1) and SK2 (KCNN2) in hair cells and efferent fiber synapses, and K_V_3.1 (KCNC1) in the spiral ganglia and ascending auditory circuits share an upregulated expression or subcellular targeting during final differentiation at hearing onset. They also share a distinctive fragility for noise exposure and age-dependent shortfalls in energy supply required for sustained surface expression. Here, we review and discuss the possible contribution of select potassium ion channels in the cochlea and auditory pathway to ARHL. We postulate genes, proteins, or modulators that contribute to sustained ion currents or proper surface expressions of potassium channels under challenging conditions as key for future therapies of ARHL.

## Introduction

Age-related hearing loss (ARHL), or presbycusis, is the most prevalent sensory deficit in the elderly [1]. Although it is not life-threatening, this condition is associated with significant psychological and medical morbidity, including social isolation, frailty, depression, and cognitive decline [2–5]. As a major risk factor for dementia [6], the prevention of hearing loss with age has been recently suggested as a foremost modifying factor to lower future dementia prevalence [7]. ARHL occurs in most mammals with variations in the age of onset, rate of decline, and magnitude of degeneration in the cochlea and the auditory pathway [8–11]. The affected cochlear structures include the stria vascularis and its vasculature, spiral ligament, sensory hair cells, and auditory neurons. Until recently, the dysfunction of the stria vascularis resulting in a reduced endocochlear potential (EP) was assumed to be a primary cause of ARHL [1, 12, 13]. However, new evidence from analyzing temporal bones of the elderly challenges this long-held view, showing that hair cell loss not only occurs in predominantly high-frequency regions but also extends to low-frequency regions in ARHL preceding stria vascularis degeneration [14]. Based on this observation, lifetime acoustic noise exposure was suggested as a primary cause of hearing loss with age, particularly due to outer hair cell (OHC) damage after acoustic overexposure, which is suggested to be the major contributor to ARHL [14]. Moreover, increasing evidence suggests that even in the absence of detectable loss of hearing sensitivity, neuronal degeneration of synaptic auditory fibers or ascending auditory projections can precede hearing threshold loss and contribute as an additional hallmark of ARHL to difficulties in speech discrimination with advancing age, especially in noisy environments [15–17]. Thus, noise exposure as a major cause of ARHL affects not only OHC over age [14] but also age-related synaptopathies and neuropathies [8], gradually leading to degeneration of spiral ganglion neurons (SGNs) [16, 18] and central auditory processing deficits [19, 20]. Furthermore, independent of lifetime noise exposure being linked to damaged hair cells and neurons, individuals with cardiovascular risk factors, e.g., hypertension, diabetes, smoking, or increased serum cholesterol, exhibit a high risk of developing hearing impairment over age [21]*.*

**Fig. 1:**
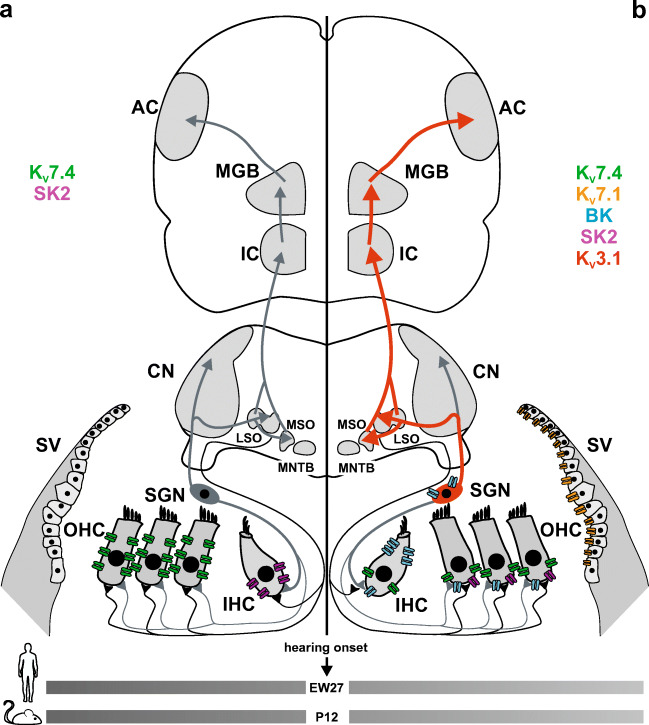
K^+^ expression along the ascending auditory pathway before and after hearing onset. **a** Before the onset of hearing, potassium ion (K^+^) channels are mainly expressed in the organ of Corti. In the outer hair cells (OHC), K_V_7.4 (KCNQ4, green) is found along the entire basolateral membrane, while the inner hair cells (IHCs) express the calcium-activated potassium channel SK2 (KCNN2, purple) before postnatal day (P) 12 in mice, corresponding to embryonic week (EW) 27 in humans. For reference, afferent (gray) and efferent (black) neural projections are shown. **b** In the mature organ of Corti, the endolymph of the scala media contains a high concentration of K^+^, which is mediated by K_V_7.1 (KCNQ1, orange) channels in the apical marginal cells of the stria vascularis (SV). During auditory stimulation, endolymphatic K^+^ enter the OHC at the basolateral membrane, and leave the cell via K_V_7.4, BK (KCNMA1, blue), and SK2 channels. In the IHC, K^+^ exits the cell through K_V_7.4 and BK channels. The expression of BK channels was identified at the lateral wall of IHC as well as in the cell bodies of spiral ganglion neurons (SGNs). The auditory signal is then transmitted from the cochlea to the cochlear nucleus (CN) via rapidly firing neurons containing K_V_3.1 (KCNC1, red arrows) channels. From here, parvalbumin-positive interneurons project onto the lateral and medial superior olive (LSO and MSO, respectively) and the medial nucleus of the trapezoid body (MNTB), whose fibers also express K_V_3.1. The inferior colliculus (IC) receives input from the contralateral (not shown) and ipsilateral superior olivary complex. The fibers from the IC project to the medial genicular body (MGB) and the signals are then transmitted to the auditory cortex (AC) via rapid firing, K_V_3.1 expressing neurons

We hypothesize that any limits in metabolic supply, e.g., from oxidative stress after acoustic trauma or limitations during ischemic insults, endanger particularly sensitive stages that require high energy supply or exhibit vulnerability for radical oxygen species (ROS) as the precursor of ARHL. Indeed, ROS contribute through reduced mitochondrial activity and enhanced oxidative damage to aging processes in all organs, and thus negatively affect hearing with advancing age [22]. We postulate that select potassium ion (K^+^) channels in the cochlea and ascending auditory pathway, which are known to critically depend on continuous recycling processes for proper surface expression, are vulnerable, early targets for limitations in energy supply. K^+^ channels show extreme genetic heterogeneity and functional diversity unmatched by other types of ion channels, which suggests them as one of the primary targets of excess ROS. Moreover, strong evidence exists that ROS-mediated oxidation of K^+^ channels is a recurring theme in the aging nervous system and is intrinsically involved in certain neuropathies [23]. Here, we focus on functionally relevant K^+^ channels in the cochlea and auditory pathway, which share common temporal expression during the final differentiation stages of the organ of Corti prior to hearing function in rodents, hypothesizing that late differentiation stages are the ones affected early during aging, offering a therapeutic window that could allow functional restoration before cell death [24]. We discuss the following K^+^ channels with functional expression during or after hearing onset, that is, around postnatal day (P) 12 in rodents, and around embryonic week (EW) 27 in humans (Fig. 1): (i) K_V_7.4 (KCNQ4) maintains OHC receptor potential [25–30]; (ii**)** K_V_7.1 (KCNQ1) is expressed in marginal cells of the stria vascularis [31–33]; (iii**)** calcium ion (Ca^2+^)-activated potassium channels BK (KCNMA1) and SK2 (KCNN2) are involved in repolarization of OHC and termination of Ca^2+^ action potential (AP) firing in medial olivocochlear (MOC) efferent fibers [34, 35]; and (iv) K_V_3.1 (KCNC1) in SGNs and ascending auditory circuits [36] are shown to be involved in temporal precision of sound processing [37]. In this review, we first summarize the expression profiles and physiological functions of these K^+^ channels, then discuss their individual roles in the context of age- and noise-dependent hearing loss, and the contribution of genetic predisposition to progressive hearing loss over age. Finally, we address respective possibilities and advantages in targeting K^+^ channels for therapeutic intervention against ARHL.

## K^+^ channels in the auditory system

### K_V_7.4 (KCNQ4)

OHC provide the mammalian ear with fast electromechanical amplification, which is required for the dynamic range and speed of sound encoding by the cochlea [38]. A direct mechanical gating of mechanoelectrical transduction channels modulates the input current at cochlear locations of sound stimulus-specific frequencies. This influx of K^+^ through apical mechanosensitive channels depolarizes the membrane and drives the contraction of OHC by the motor protein prestin [39]. The speed of this action depends on the capacitance and conductance of the OHC at resting membrane potential, which in turn critically depend on determinants of OHC conductance maintained through the efflux current I_K,n_, mediated by the voltage-gated potassium channel subunit K_V_7.4 [40].

While K_V_7.4 expression [41, 42] and its current I_K,n_ [40] are detected prior to hearing onset along the entire basolateral membrane of OHC in mice (Fig. 1a), K_V_7.4 is redistributed after the onset of hearing (P12-14), becoming restricted solely to the basal pole (Fig. 1b) [43, 44]. This localization suggests that K_V_7.4 serves to extrude K^+^ ions that enter OHC through the apical mechanosensitive channels [28, 29, 45, 46]. K_V_7.4 is also detected in inner hair cells (IHCs) [25, 26], SGNs, and several nuclei along the auditory pathway, e.g., cochlear nuclei and inferior colliculus [25, 29].

Impaired surface expression of K_V_7.4 in hair cells has been shown to be a primary step of hearing loss [47–50]. In *Kcnq4* knock-out mice, Carignano et al. [49] showed that the number of OHC slowly decreased at a young age with increasing cell loss up to complete degeneration at oldest ages. Degeneration of IHCs was also observed, but only in the adult stage. The loss of this important K^+^ channel in OHC results in a chronic depolarization, possibly increasing Ca^2+^ influx through voltage-gated Ca^2+^ channels and causing their subsequent degeneration due to chronic cellular stress [51].

### K_V_7.1 (KCNQ1)

The sensory cells of the inner ear are in contact with the fluids in the scala media which is filled with endolymph, the extracellular fluid with high K^+^ concentration. K^+^ is the major charge carrier for sensory transduction and its proper circulation is of great importance for the process of hearing. K^+^ ions are secreted into the endolymph by the stria vascularis, enter the hair cells through apical mechanosensitive non-selective cation channels, and exit these cells via their basolateral membrane, then migrate through supporting cells and fibrocytes towards the stria vascularis using a network of gap junctions where they are reabsorbed by strial marginal cells and released into the endolymph [52, 53]. K_V_7.1 (KCNQ1) and its β-subunit KCNE1 form a channel complex that is expressed in the mature organ of Corti in the apical membrane of marginal cells of the stria vascularis where it mediates the slow delayed rectifier current I_K,s_ [31–33, 54]. As components of K^+^ circulation, K_V_7.1 and KCNE1 are responsible for the secretion of potassium to the endolymph [55, 56], generating the EP [57, 58].

K_V_7.1 is expressed throughout the body including the liver, lung, heart, and cochlea [31–33]. The homomeric form of K_V_7.1 gives rise to a slowly activating and deactivating voltage-dependent potassium current [33]. However, in the inner ear, K_V_7.1 modulates the kinetics by assembling to KCNE1 to form a heteromeric channel [32]. This results in a drastic slowdown in channel activation, a positive shift in voltage activation threshold, and an absence of inactivation [31]. During cochlear development, K_V_7.1 was not detected at several embryonic stages in mice (Fig. 1a), indicating that its expression is first established during the postnatal stages (Fig. 1b) [59].

Loss of functional K_V_7.1 or KCNE1 leads to Jervell and Lange-Nielsen syndrome which is characterized by cardiac arrhythmia [60–63] and associated with congenital deafness in humans [32, 62, 64, 65]. Potassium secretion into the endolymph is consequently disturbed causing a defect of endolymph production and a collapse of the Reissner membrane [66].

### BK (KCNMA1) and SK2 (KCNN2)

Calcium-activated potassium channels are divided into two broad categories, small conductance calcium-activated SK channels and large conductance, voltage-gated, and calcium-sensitive BK channels [67]. SK channels have high Ca^2+^ affinity and long open times, while BK channels are distinguished by significant differences in voltage sensitivity, single-channel conductance, Ca^2+^ affinity, and gating kinetics [68]. These channels share the common functional role of coupling the increase in intracellular Ca^2+^ concentration to hyperpolarization of membrane potential, thus playing an important role in cellular excitability and maintaining K^+^ homeostasis [69].

Calcium-activated K^+^ conductance has been described in both OHC and IHCs [45, 70]. BK decreases membrane time constants and enables the fast repolarization of hair cell receptor potentials [46] and efferent fibers [71]. BK channels in hair cells appear to show tonotopic gradients of increasing expression from apical (low frequency) to basal (high frequency) regions [34, 35, 72]. The stronger expression in high-frequency regions suggests a contribution of BK channels to high-frequency hearing in mammals. Furthermore, application of acetylcholine, a major efferent neurotransmitter, has been shown to exclusively activate BK currents in high-frequency OHC as opposed to SK currents in the lower-frequency OHC [35], and has been shown to modify efferent inhibitory synaptic responses in high-frequency OHC [73].

In the developing mouse, SK2 channel expression in IHCs was demonstrated during the first two postnatal weeks with a peak around P9 (Fig. 1a), disappearing during hearing onset with decline of cholinergic axosomatic efferent IHC innervations (Fig. 1b) [74]. BK channel expression has been identified in the cell bodies of SGN as well as in inner and outer sensory hair cells at the onset of hearing around P12 (Fig. 1b) [75, 76]. The appearance of the fast BK current, I_K,f_, in IHCs has been shown to coincide with the disappearance of spontaneous action potentials, transforming mature mammalian IHCs into high-frequency signal transducers [77, 78]. During the first four postnatal weeks, BKα^−/−^ mice surprisingly did not show any obvious hearing deficits [51]. High-frequency hearing loss developed in BKα^−/−^ mice only from approximately 8 weeks postnatally onward and was accompanied by a lack of distortion product otoacoustic emissions, suggesting OHC dysfunction.

### K_V_3.1 (KCNC1)

The *Kcnc1* gene yields two K_V_3.1 subtypes (a and b) through alternative splicing [79], but K_V_3.1b has been shown to predominate in the adult rodent brain [80, 81]. Apart from the medial nucleus of the trapezoid body (MNTB) and anteroventral cochlear nucleus (AVCN), K_V_3.1 is also expressed in neurons of the reticular thalamic nucleus and parvalbumin-positive (PV+) interneurons of the cortex and hypothalamus [81, 82]. K_V_3.1 belongs to the delayed rectifier channel family and is located on presynaptic terminals [83–85]. Its high activation threshold and rapid activation and deactivation in response to voltage changes reduce the AP duration while simultaneously maximizing firing frequency [86]. This special characteristic of K_V_3.1 for maximizing firing frequencies is related to its distinct expression profile in fast spiking interneurons [81, 82] and the important role it plays in the auditory system.

During auditory pathway maturation, K_V_3.1 levels increase in SGNs between P4 and P8, reducing AP latencies and duration after hearing onset [87, 88]. The expression level of K_V_3.1 rises dramatically near the onset of hearing along with the maturation of fast auditory processing as shown in the brainstem [89–91] and the inferior colliculus (Fig. 1b) [92, 93]. This expression profile in fast PV+ interneurons makes K_V_3.1 a key contributor to the lowered threshold and shortened latency of cortical auditory responses, which can be measured after the sharpening of cortical receptive fields [94] at the end of the critical period after hearing onset. Thus, receptive field maturation coincides with the maturation of a network of fast-spiking GABAergic PV+ interneurons [95–98], predicted to mature in the auditory pathway with fast auditory processing after hearing onset [99]. Accordingly, given the optimal design of K_V_3.1 for high-rate repetitive firing [100, 101], it has been identified as critical for fast-spiking PV+ interneurons [102]. Also, the key components in the auditory pathway required for auditory discrimination, the MNTB and AVCN, contain neurons that fire at very rapid rates, requiring the expression of K_V_3.1 for rapid repolarization of AP during sound-induced activity [103–105]. MNTB neurons of K_V_3.1 deficient mice were incapable of following high-rate stimulation or sustaining high-rate firing AP [37], demonstrating that K_V_3.1 is essential for the rapid firing patterns. Given that hearing impairment can lead to a decline in K_V_3.1 expression in the MNTB [36, 106], it is likely that the lack of K_V_3.1 channels is a key contributor to deficits in fast auditory discrimination over age [107].

## Noise exposure linked to ARHL

The driving mechanisms of hearing loss over age remain largely unclear. Already in rodent animal species that are widely used as models for human hearing, the age-related loss of cochlear function is highly variable; different mouse lines display hearing loss as early as 5 weeks after birth, determined partly by species, strain, and animal history, but also partly by lifetime auditory exposure determined by noise intensity level, duration, predictability, exposure context, and other characteristics of the sound [108]. In healthy-aged Mongolian gerbils, auditory-evoked potentials show a decrease of responses before the loss of auditory sensitivity, which is attributed to age-related pathologies in the auditory periphery [109, 110]. Studies in quiet-aged gerbils suggest that loss of synapses is the earliest age-related degenerative event (reviewed in [111]), preceding strial dysfunction and other cochlear pathologies [112]. Functional studies on aging rats have confirmed this [113] and extended the functional consequences of the loss of synapses beyond hearing sensitivity towards the loss of central compensatory action of the brain to make use of the few remaining auditory signals. In the cochlea, aging in gerbils and rats is characterized by threshold increase and concurrent loss of normal OHC phenotype from the second third of their lifespan onwards, which is related to a reduced brain-derived neurotrophic factor (BDNF) expression levels in the auditory nerve [114].

In CD-1 mice, often used as a model for human hearing, the EP is already lost at the age of 9 months, and the sensory organ is completely degenerated [115]. CBA/CaJ mice are described to have normal EP and excellent hearing for a large portion of their lifespan. Nevertheless, they display a remarkable acceleration of ARHL when repeatedly exposed to “benign” noise during their lifespan [116, 117]. By contrast, 129/SvEv mice are exceptionally resistant to noise-induced hearing loss [118], but preexisting anomalies in substrains of 129/SvJ mice predispose the ear to degenerate prematurely when interacting with K^+^ channel deletion [118]. Finally, ROS-induced activation of DNA damage in senescence-accelerated mouse-prone 8 (SAMP8) mice are discussed as the driver for ARHL [119]. ROS can be induced in the ear by exposure to moderate, nevertheless harmful, acoustic noise [120, 121] causing an accumulation of toxic noise events throughout lifetime (reviewed in [122]). We have to assume that even the early loss of synaptic contacts between sensory hair cells and SGNs or synaptopathy [17] can be traced back to cumulative excitotoxic injury events [123], the largest source of which is likely to be noise exposure [124, 125].

One of the earliest events following metabolic limitations during noise exposure is the impairment of membrane surface expression of distinct K^+^ channels in the cochlea, a process that is here suggested to have a pivotal role in ARHL. Both K_V_7.4 (KCNQ4) and BK (KCNMA1) channels are required for normal hearing and have been suggested to protect OHC in cochlear regions that register high frequencies from Ca^2+^ overload [47, 72]. Functional loss in OHC has been linked to the loss of K_V_7.4 in the membrane of the OHC, preceding their degeneration in the middle- and high-frequency coding cochlear compartments [51, 126]. The loss of BKα led to a similar phenotype as by pharmacological blockage of K_V_7.4 channels, suggesting that a loss of the *BK* gene increases susceptibility for progressive ARHL, similar to *KCNQ4* mutation [26, 47, 51, 78]. Consistent with that assumption, exposure to a low-frequency, non-traumatic sound has been found to not affect hearing sensitivity of wild-type mice, but mice with *BKα* gene deletion experienced a dramatic loss of hearing sensitivity within the stimulated low-frequency hearing range [72]. It is important to note that the affected low-frequency range was not part of the hearing range affected by accelerated ARHL in the young unexposed BKα deficient mice, thus confirming that the low-frequency cochlear compartments are rendered susceptible by the absence of BK. The low-frequency noise exposure extended the loss of KCNQ4 from OHC towards the low-frequency cochlear compartments affected by the noise exposure, confirming that the hearing loss resulted from the absence of KCNQ4 from hair cell plasma membrane [72]. The metabolic balance due to the fast repolarization of the receptor potential is a requirement for the healthy homeostasis of hair cells. Thus, the maintenance of KCNQ4 and BK in the OHC membrane is most critical to counteract a *Ca*^*2+*^ overload of hair cells, irrespective of whether induced through excitotoxic, ototoxic, or noise exposure events, all of which are challenges that accumulate over advancing age. The activity in MOC efferent fibers contacting OHC plays an important role to activate BK and SK2 channels through acetylcholine release. Therefore, any reduction in MOC efferent fibers, which were previously shown to decline with advancing age [127], is expected to increase susceptibility to noise-induced hearing loss over age, due to reduced potential to rapidly remove K^+^ from OHC, as can be predicted from various studies [34, 51, 72, 128, 129].

Furthermore, the protective role of BK channels is not limited to the hearing organ. In the mammalian central nervous system, BK is expressed in the neuron soma, processes, and presynaptic terminals, where it drives the membrane potential towards the potassium equilibrium potential to re- and hyperpolarize the neuron [130]. To study the importance of central BK deletion in the brain, normal hearing mice are required. Fortunately, the F1 generation of a hybrid sv129/C547/Bl6 background of mice with genetic deletion of the BK channel has documented good hearing up to the age of 15 weeks [131], which again confirms the multifactorial nature of BK gene deletion-related progression of hearing disorder. Most strikingly, these mice nevertheless display slower learning capacity and no improvement of pre-pulse inhibition of the acoustic startle response over days [131]. This strongly suggests that besides the protective role of cochlear BK channels [51, 72], they further contribute to the integrity of central neuronal circuits that are essential to process environmental auditory information. Because the top-down modulation of cochlear hair cells’ excitability is assumed to play a critical role in adaptation to and avoiding damaging influences of environmental changes [132–134], the centrifugal control of neuronal excitability may be a major factor of K^+^ channel-related ARHL.

SK2 (KCNN2) is expressed in OHC as a postsynaptic marker apposing synapses of MOC efferent fibers and required for Ca^2+^-activated SK2 channel activation through MOC’s cholinergic function [135]. The number of SK2-positive foci is remarkably reduced in mouse strains that exhibit fast progression of ARHL, such as the C57BL/6J [136], thus representing a general trait in the pathophysiological progression of ARHL. The protective role of MOC efferent fibers during aging and noise only recently received support through the discovery that loss of MOC efferent fibers is an early event of ARHL [127]. We may thus conclude that loss of BK, SK2, and KCNQ4 is likely to be early contributors to ARHL, with their dysfunction discussed as a primary event of OHC loss over age.

K_V_7.1 (KCNQ1) is a major component of the K^+^ circulation by the stria vascularis and is responsible for the secretion of potassium to the endolymph and maintaining the EP, to assist motility in OHC, perform synaptic activity, and maintain the spontaneous and evoked activity of SGNs. The cells of the stria vascularis contain high numbers of mitochondria [137, 138] and Na^+^/K^+^ATPase [139, 140]. In quiet-aged gerbils, the stria vascularis and spiral ligament showed a decrease in Na^+^/K^+^ATPase activity in these tissues [141], as well as degeneration of strial capillaries at both ends of the cochlear spiral [142] and decreased blood flow [143]. In aged CBA/CaJ mice, Na^+^/K^+^ATPase expression was largely reduced, and the stria vascularis was found to be atrophied [139]. However, it remains difficult to determine whether the lack of blood flow or the cellular dysfunction leads to the strial atrophy. As such, most of these studies did not specifically analyze membrane expression patterns of KCNQ1 during stria vascularis degeneration over age. Interestingly, 12-month-old C57BL/6 mice displayed notable hearing loss and morphological examination showed a significant OHC loss in the cochlear basal turn accompanied by atrophy of the stria vascularis, with immunohistochemical analysis revealing dramatically decreased KCNJ10 and KCNQ1 expression [144]. While these studies observed a conservation of the EP in these aging C57BL/6 mice, and suggested that the stria vascularis can generate a new balance for potassium influx and efflux at relatively low turnover [144], other studies found a clear requirement of adequate KCNQ1 recycling in marginal stria vascularis membranes for hearing and OHC cell survival [145]. On the whole, age-dependent decline of KCNQ1 from the marginal surface of the stria vascularis should be urgently reconsidered with regard to ARHL.

The expression of K_V_3.1 (KCNC1) subtype b, which is predominant in the adult rodent brain [80, 81], has been shown to decrease in neurons of the MOC efferent system by middle age in CBA/CaJ mice, and these changes appeared to correlate with functional declines in efferent activity in both middle-aged CBA/CaJ mice and K_V_3.1b knockout mice [106, 146], suggesting age-dependent decline of K_V_3.1b as a possible cause of MOC efferent decline over age [127]. Also, C57BL/6 mice have been shown to lose sensory basal hair cells during early adulthood progressing towards the apex with age [147], which was linked to a concurrent decrease in levels of K_V_3.1b in brainstem neurons [106]. In Sprague-Dawley rats, both the intensity of K_V_3.1 immunostaining and number of K_V_3.1-positive neurons have been shown to decline with age in the cochlear nucleus [148]. Age-dependent K_V_3.1 modifications are expected to contribute to age-dependent temporal discrimination deficits [149]. This is particularly important when considering the special role K_V_3.1 activity displays as a modulator for fast-spiking inhibitory PV+ interneurons, which control feedforward and feedback inhibitory modalities [150], suggested to be essential for fast auditory processing circuits [99]. Reduced PV expression levels have also been found in the auditory cortex in aged animal models [151], which implies a potential relation between the decline of K_V_3.1 expression over age and PV-mediated processing deficits in ARHL.

**Fig. 2:**
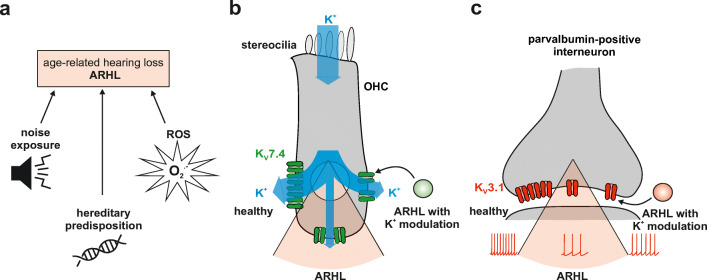
**a** In the challenged auditory system leading to age-related hearing loss (ARHL), the key causing factors are postulated to be lifetime noise exposure, hereditary predisposition, and the accumulation of reactive oxygen species (ROS). **b** In a healthy outer hair cell (OHC), potassium ions (K^+^) enter the cell through apical mechanosensitive channels and are then transported to the supporting cells through K_V_7.4 (KCNQ4, green) channels on the basolateral membrane of OHC. However, in the challenged system, the expression of K_V_7.4 is reduced, resulting in a poor K^+^ efflux. This state can be influenced by the addition of K^+^ channel modulators (green circle) in a way where cell surface expression remains stable but the efflux rate can be increased. **c** In fast-spiking, parvalbumin-positive interneurons, K_V_3.1 (KCNC1, red) is required for the high-frequency repetitive firing. A decline in K_V_3.1 cell surface expression leads to an incapacity of neurons to maintain high-frequency firing action potentials. Modulators that bind to K_V_3.1 (red circle) may lower action potential latencies and duration and increase the firing pattern of these neurons

Based on evidence from different animal models and from human temporal bones, it seems likely that aging or senescence alone is not necessarily a major risk factor for hearing impairment over advancing age. Convincing evidence that aging per se is not necessarily the main cause for ARHL comes from geriatric cats that have normal hearing sensitivity and auditory brainstem functions over the whole frequency range of hearing, developing ARHL only late in their lifespan [152]. The current evidence suggests that an accumulation of noise events may most likely be the origin of ARHL in humans (Fig. 2a), with excessive noise able to overstimulate sensory hair cells and requires fast and effective K^+^ recycling within the inner ear. This suggests that ARHL may rather be an accumulation of damage from minor toxic events instead of an inevitable progressing loss of cells, structures, and the ability for regeneration.

## Genetic predisposition to ARHL

The human genome contains roughly 70 K^+^ channel-encoding genes [153]. Many of these genes play essential roles in normal physiological processes, including those involved in hearing, as evidenced by the clinical appearance of syndromic and/or non-syndromic hearing loss with genetic variation. As ARHL has a delayed onset and progressive nature, it is plausible to hypothesize that genetic variation may directly contribute to an inter-individual variability and susceptibility to ARHL or to other indirect processes of aging such as metabolic status. One way to test this is to perform statistical analyses in the form of association testing to identify genetic regions or variants, in either genes of interest or across the entire genome, to identify associations with ARHL. This type of analysis has not been performed for all of the genes encoding the channels discussed in this review. However, in the following section, we summarize the current body of genetics knowledge for our selected K^+^ channels and make connections to ARHL.

The *KCNQ4* gene encodes the potassium voltage-gated channel subfamily Q member 4 protein (K_V_7.4). Deleterious variants in *KCNQ4* cause autosomal dominant non-syndromic hearing loss (MIM* 603537) [30]. A significant association between *KCNQ4* and ARHL has been identified by Van Eyken et al. [154] in two independent populations. However, except for one single nucleotide polymorphism (SNP), i.e., SNP12 (rs2149034), different SNP spanning a 13-kb region of *KCNQ4* were positively associated in both populations. *KCNQ4* was regarded by the authors as a strong susceptibility gene for ARHL; however, replication studies have not reproduced this observation. *KCNQ4* expression increases with age, supporting a hypothesis that an increased defective protein load may lead to progressive cellular dysfunction [154, 155]. Van Laer et al. [156] also found significant differences between individuals susceptible and resistant to noise exposure for the allele, genotype, and haplotype frequencies for a *KCNQ4* SNP (rs34287852). A genome-wide association study (GWAS) meta-analysis from the Cohorts for Heart and Aging Research in Genomic Epidemiology or CHARGE Consortium was performed with the aim to identify genetic factors associated with overall mortality and healthy longevity [157]. This study identified 14 independent SNP that predicted risk of death and eight that predicted healthy longevity. Several of these SNP were located either in or near genes that are involved in neurological processes. A *KCNQ4* SNP (rs2769255) was significantly associated with both mortality and healthy longevity and is located approximately 4.4 kb upstream from the gene. The enrichment of SNP, either in or adjacent to genes involved in neurological processes, suggests the importance of these genes in regulating healthy aging and longevity.

The *KCNQ1* gene encodes the potassium voltage-gated channel subfamily Q member 1 protein (K_V_7.1). *KCNQ1* variants have been associated with long QT syndrome, short QT syndrome, atrial fibrillation, and Jarvell and Lange-Nielsen syndrome (MIM* 607542) [158]. The same study by Van Laer et al. [156] also found one significant difference between noise susceptible and resistant individuals in one *KCNQ1* SNP (rs163171). The most interesting literature linking KCNQ1 to ARHL comes from the diabetes field, which constitutes a risk factor for ARHL [21]. With respect to a broader biological context, *KCNQ1* is also expressed in the heart, stomach, intestine, liver, kidney, and insulin-producing cells [159, 160]. Several GWAS have uncovered many independent intronic regions in *KCNQ1* that harbor type 2 diabetes mellitus risk alleles (rs231362, rs2283228, rs2237892, rs2237895, and rs2237897) in Europeans, East Asians, and Native Americans [161–164]. It is unclear if the SNP exert a functional effect and whether this would involve *KCNQ1* or the neighboring genes, *KCNQ1OT1*, or *CDKN1C*, a known regulator of pancreatic beta-cell development [163]. Neither *Kcnq1* null mice nor patients with deleterious variants show impaired hyperglycemia or glucose intolerance; therefore, it is thought that an increase in expression in pancreatic beta-cells may be linked to the development of type 2 diabetes [163]. Interestingly, *KCNQ1* resides on chromosome 11p15.5, a maternally imprinted region [161]. This means that maternally inherited variants in this imprinted region confer disease risk. There is compelling evidence that diabetes risk at the *KCNQ1* locus is medicated through a gene with imprinted gene expression that may be mediated by *KCNQ1* or neighboring genes (*KCNQ1OT1* or *CDKN1C*) [165]. The confirmation of so-called parent-of-origin effects that have been identified in every organ system of the human body so far except the auditory system would fundamentally re-shape the way the genomics field views genetic contributors of ARHL [166].

The *KCNMA1* (BK), *KCNN2* (SK2), and *KCNC1* (K_V_3.1) genes presently do not have compelling evidence linking them to ARHL, but several already have gene-disease associations. The *KCNMA1* gene encodes the potassium calcium-activated channel subfamily M alpha 1 protein (BK). Deleterious *KCNMA1* variants are associated with paroxysmal nonkinesigenic dyskinesia with or without generalized epilepsy, Liang-Wang syndrome and cerebellar atrophy (MIM* 600150). Although no current genetic-based studies linking *KCNMA1* SNP to presbycusis and aging exist, BK channels appear to be sensitive to oxidative stress [167]. The *KCNN2* gene encodes the potassium calcium-activated channel subfamily N member 2 protein (SK2) and is currently not associated with any human phenotypes. The SK channel has been linked to neuroprotection in the form of mitochondrial resilience against neuronal death [168]. SK channels may involve attenuation of mitochondrial calcium uptake upon SK channel activation. Mitochondrial activation calcium uptake across the mitochondrial membrane is essential for the numerous calcium-sensitive processes required for mitochondrial metabolism and respiration [169]. Oxidative stress in neurons leads to a series of detrimental effects such as intracellular calcium overload that induces changes in mitochondrial metabolism such as alterations in ATP synthesis and NADP(H) oxidation that lead to an increase in ROS [168, 170]. Finally, the *KCNC1* gene encodes the potassium voltage-gated channel subfamily C member 1 protein (K_V_3.1). It has been associated with autosomal-dominant progressive myoclonic epilepsy (MIM* 176258) [171] and intellectual disability without seizure or epilepsy [172].

On the whole, the current literature lacks conclusive human genetic evidence to link ARHL and *KCNQ1* (K_V_7.1), *KCNMA1* (BK), *KCNN2* (SK2), and *KCNC1* (K_V_3.1), but contains limited information to link it to *KCNQ4* (K_V_7.4). These genes are still intriguing due to possible gene-environment interactions in processes such as aging and metabolism that are presently unknown (Fig. 2a). ARHL is a complex disorder with environmental, e.g., noise exposure, and genetic factors. Twin studies for ARHL have estimated the heritability of ARHL, or importance of a genetic component in a disease [154] and found that twin similarity of monozygotic twins decreased with age and dizygotic twins increased with age [173]. This suggests that environmental factors may become more prominent with age. Of note, SNP in K^+^ channel genes have not been noted with significance in the more recent large-scale genomics studies [174, 175]. However, if K^+^ channel genetic targets are identified with future ongoing studies, they have the potential to make excellent therapeutic targets.

## K^+^ channels as therapeutic targets against ARHL

Having given a comprehensive view about the role select potassium channels play in the cochlea and the ascending auditory pathway for ARHL in the context of noise exposure and genetic predispositions, we may next illuminate therapeutic intervention strategies with a potential to prevent or repair hearing dysfunction as future ARHL therapies. Provided that noise exposure, age-related synaptopathies and neuropathies, and cardiovascular risk factors are major contributors of ARHL [8, 14, 21], substantial evidence points to oxygen metabolism as one of the main culprits for K^+^ channel dysfunctions with aging given that these dysfunctions are not only based on channel mutations. Numerous studies evidenced that ROS increases with age [176] and by statistical probability alone preferentially affects K^+^ channels (Fig. 2a). The extreme genetic heterogeneity and functional diversity of K^+^ channels are unparalleled to that of other types of channels [23]. ROS can indirectly modulate K^+^ channel function by acting on cellular pathways that regulate gene transcription, trafficking, turnover, and proteasomal degradation [177]. On the other hand, direct age-related oxidation of particular voltage-dependent K^+^ channels that include the aforementioned K_V_7 channels, Ca^2+^-activated BK and SK2, and K_V_3.1 underlie a specific type of neuronal aging [23]. In the auditory system, several lines of evidence hint to the importance of these channels with respect to ARHL as discussed above.

*KCNQ* genes have a considerable physiological impact in many cell types. This reliance upon K_V_7 channels for normal cellular function is evident by hereditary disorders caused by mutations in *KCNQ* genes, meaning that pharmacological targeting of these channels has broad appeal. Consequently, a plethora of chemical entities that modulate K_V_7 channel activity has been developed. Moreover, K_V_7 channels are influenced by many disparate intracellular mediators and trafficking processes, making upstream targeting an appealing prospect for therapeutic development to overcome deficits related to these channels [178]. Until now, however, modulation of K_V_7 channels has been recognized mainly as a potential to prevent neurodegenerative disorders linked to epilepsy and cognitive deficits [179]. Although efforts have not reached ARHL, pharmacological approaches in trials targeting K_V_7.2 to K_V_7.5 channels with the novel antiepileptic drug retigabine (or ezogabine) have been used to overcome hearing loss [180, 181]. Retigabine increases the probability of opening these K_V_7 channels upon causing a negative 15-mV leftward shift in the voltage-dependence of activation and a decrease in the rate of deactivation (Fig. 2b) [178, 182–184].

Given a hereditary origin of progressive hearing loss through *KCNQ4* dysfunction, as it occurs in DFNA2 non-syndromic autosomal-dominant progressive high-frequency hearing loss [155, 157], genetic therapeutic approaches have been envisioned, e.g., those following heterologic expression of wild-type channels that could be combined with K_V_7 channel openers such as retigabine [181]. Correspondingly, retigabine has already proven successful to rescue hearing deficits in Korean families with co-segregating *KCNQ4* pathogenic variants [180]. Also, a combination of zinc pyrithione plus retigabine has been used in Chinese hamster ovary cells either transfected with wild-type *Kcnq4* sequences or ones containing variants that encode mutated channels, evidencing a restoration of channel function that was dependent on the location of the DFNA2 mutation within the gene [185]. This further provides an interesting approach to rescue progressive ARHL linked with mutations of *KCNQ* genes on the personalized medicine level.

Undoubtedly, K_V_7.1 (KCNQ1) expression decreases with advancing age in the stria vascularis [144], but, as previously highlighted, it may only contribute to ARHL in a secondary manner [14]. Hormone changes may be considered as contributors to the decline of K_V_7.1 surface expression loss in the stria vascularis with age. Thus, throughout the lifespan, the steroid hormone estrogen (17β-oestradiol, E2) declines with age in females [186]. Estrogen decline has been suggested to induce K_V_7.1 dysfunction through changes in estrogen-dependent control of its internalization from the plasma membrane by a clathrin-mediated endocytosis process [187]. Estrogen has been shown to modulate the association between K_V_7.1 and the clathrin adaptor AP-2, required for endocytosis, rather than degrading the ion channel, and a biphasic recycling mechanism involving Rab4 and Rab11 is involved in this process, as shown in colon epithelium [187]. Modulators of K_V_7.1 may thus contribute to overcome postmenopausal-related hearing loss reported to occur with aging [188].

Within this context, it may be interesting to note that a spatio-temporal correlation of the loss of KCNQ1 and KCNE1 surface expression and loss of hearing thresholds has been reported following loss of proteins involved in KCNQ1 recycling, such as SCARB2 [145]. Human SCARB2 is a key regulator of lysosome integrity, motility, and dynamics, and its loss has been shown to cause rupture of lysosome membranes and significantly shortened lifespan [189]. This may suggest any disturbance of proper membrane recycling or insufficient targeting of KCNQ1 and KCNE1 in the stria vascularis, might be a possible rationale for ARHL [190], and renders lysosomal enzymes that stimulate trafficking as potential candidates for targeting ARHL [191].

The Ca^2+^-activated channels BK (KCNMA1) and SK2 (KCNN2) play an important role in noise-induced ARHL, counteracting noise-induced hyperpolarization of OHC. These may be particularly sensitive for age-dependent ROS damage, being both susceptible to redox modifications [23, 192]. The noise-induced rise in Ca^2+^ in OHC (i.e., Ca^2+^ overload) is expected to induce slow cellular after-hyperpolarizations for SK2 and fast ones for BK channels, both possibly contributing to the prevention of noise damage to OHC [34]. Within this context, the specific role of BK in IHCs, shown to rapidly and robustly shape IHC receptor potential [193], needs to be considered. An oligonucleotide antisense against SK channels was shown to compensate an age-related memory decline in mice, resulting from ROS-induced modification of SK channel function [194, 195], providing viral-mediated expression of SK2 channel as a potential means to target its deficits with advancing age. For BK channels, specific blockers have been shown to counteract the negative redox effects in the brain, indicating that ROS-signaling on BK channels leads more to active, rather than inactive, channels [196]. This would expectedly lead to reduced neuronal excitability of hair cells, through upregulation of K^+^ channel activities as a hallmark of the aging process. This hypothesis for the aging cochlea awaits further studies and requires reflection in the context of age-dependent deficits of fast auditory processing [197, 198].

A-type voltage-gated potassium (K_V_) channels, to which the K_V_3.1 (KCNC1) channel belongs, are sensitive for age-dependent ROS changes [199], resulting through oxidation of channels in slowed inactivation and increased open channel currents, modifications that would dampen neuronal excitability as shown for the hippocampus [200, 201]. K_V_3.1 has not only been shown as important for sustained temporally accurate firing, being susceptible to deprivation, but also to its deficits partially restored in animals by the compound AUT00063 (Fig. 2c) [197]. AUT00063 has also been shown to improve auditory synchronization and support more accurate decoding of temporal sound features in the inferior colliculus and auditory cortex in adult mice with a near-complete loss of auditory nerve afferent synapses in the contralateral ear [197], rendering K_V_3.1 modulators an attractive candidate for pharmaceutical targeting against fast auditory processing deficits due to ARHL. Furthermore, antidepressants, such as p11, have been shown to control K_V_3.1 expression level and intracellular localization in PV+ interneurons of the hippocampus [202]. With reduced K_V_3.1 levels, the capacity of PV+ interneurons to adapt to high-frequency firing is abolished [202], underscoring the crucial role that sustained expression levels of K_V_3.1 may have over age for preserving temporal auditory processing and speech discrimination. Importantly, the high metabolic vulnerability of particular PV+ interneuron synapses [203] should be reconsidered in the context of required sustained K_V_3.1 channels for its proper function in the ascending auditory pathway. K_V_3.1 channel modulators have recently been shown to enable faster activating kinetics and increase firing frequency in fast-spiking GABAergic interneurons [204, 205]. This renders these modulators as promising candidate pharmaceutical drugs to overcome ARHL, with a potential to improve speech in noise deficits, especially with regard to the reconsidered role that maintained PV+ interneuron-mediated feedforward and feedback circuits have in fast auditory processing [99].

## Outlook

In humans, the classification of various presbycusis profiles over age is manifold, but despite profound heterogeneity, most of the presbycusis profiles are characterized by a dominant loss of sensitivity to high-frequency tones [206]. Therefore, loss of auditory sensory function with age must be classified by the probable excessive noise exposure as a main contributor [14]. The current review suggests that noise-induced overstimulation of sensory hair cells and neurons most critically depends on fast and effective K^+^ recycling in the cochlea, including sustained fast auditory processing that may be required for K_V_3.1-driven, fast PV+ interneuron function over age. Pharmaceutical targeting of K^+^ channels to enable fast recycling through stimulators, modulators, or activators has future potential to arrest or even prevent ARHL before the inevitable progression of loss of cells, structures, and degeneration.

An important caveat to consider with respect to different functional consequences of oxidation for the reviewed K^+^ channels is the rationale against considering therapies based on generic anti-oxidants for the treatment of ARHL. Modes of interventions aimed at targeting more specific channel proteins or distinctly responsible ROS species, which is not a simple task, may be more likely to succeed. The ability to pharmacologically separate the impact of individual K^+^ channel subunits needs further refinement, beginning with existing compounds and reinforcement with molecular interference techniques.

Although many clinicians inform patients that ARHL cannot be prevented, animal model studies provide insight and future prospects for clinical trials and even clinical interventions to prevent or slow the progression of ARHL.

## References

[1] Bowl MR, Dawson SJ (2019) Age-related hearing loss. Cold Spring Harb Perspect Med:9. 10.1101/cshperspect.a033217 10.1101/cshperspect.a033217 PMC6671929 30291149

[2] Lin FR, Yaffe K, Xia J, Xue QL, Harris TB, Purchase-Helzner E, Satterfield S, Ayonayon HN, Ferrucci L, Simonsick EM, Health ABCSG (2013). Hearing loss and cognitive decline in older adults. JAMA Intern Med.

[3] Kamil RJ, Betz J, Powers BB, Pratt S, Kritchevsky S, Ayonayon HN, Harris TB, Helzner E, Deal JA, Martin K, Peterson M, Satterfield S, Simonsick EM, Lin FR, Health ABCs (2016). Association of hearing impairment with incident frailty and falls in older adults. J Aging Health.

[4] Rutherford BR, Brewster K, Golub JS, Kim AH, Roose SP (2018). Sensation and psychiatry: linking age-related hearing loss to late-life depression and cognitive decline. Am J Psychiatry.

[5] Lin FR, Ferrucci L, Metter EJ, An Y, Zonderman AB, Resnick SM (2011). Hearing loss and cognition in the Baltimore Longitudinal Study of Aging. Neuropsychology.

[6] Livingston G, Sommerlad A, Orgeta V, Costafreda SG, Huntley J, Ames D, Ballard C, Banerjee S, Burns A, Cohen-Mansfield J, Cooper C, Fox N, Gitlin LN, Howard R, Kales HC, Larson EB, Ritchie K, Rockwood K, Sampson EL, Samus Q, Schneider LS, Selbæk G, Teri L, Mukadam N (2017). Dementia prevention, intervention, and care. Lancet.

[7] Montero-Odasso M, Ismail Z, Livingston G (2020). One third of dementia cases can be prevented within the next 25 years by tackling risk factors. The case “for” and “against”. Alzheimers Res Ther.

[8] Fischer N, Johnson Chacko L, Glueckert R, Schrott-Fischer A (2020). Age-dependent changes in the cochlea. Gerontology.

[9] Frisina RD (2001). Subcortical neural coding mechanisms for auditory temporal processing. Hear Res.

[10] Frisina RD (2009). Age-related hearing loss: ear and brain mechanisms. Ann N Y Acad Sci.

[11] Frisina RD, Frisina DR (2013). Physiological and neurobiological bases of age-related hearing loss: biotherapeutic implications. Am J Audiol.

[12] Ohlemiller KK (2004). Age-related hearing loss: the status of Schuknecht’s typology. Curr Opin Otolaryngol Head Neck Surg.

[13] Merchant SN, Nadol JB (2010) Schuknecht’s pathology of the Ear. People’s Medical Publishing House-USA

[14] Wu PZ, O’Malley JT, de Gruttola V, Liberman MC (2020). Age-related hearing loss is dominated by damage to inner ear sensory cells, not the cellular battery that powers them. J Neurosci.

[15] Plack CJ, Barker D, Prendergast G (2014) Perceptual consequences of “hidden” hearing loss. Trends Hear 18. 10.1177/2331216514550621 10.1177/2331216514550621 PMC4227662 25204468

[16] Kujawa SG, Liberman MC (2015). Synaptopathy in the noise-exposed and aging cochlea: primary neural degeneration in acquired sensorineural hearing loss. Hear Res.

[17] Wu PZ, Liberman LD, Bennett K, de Gruttola V, O’Malley JT, Liberman MC (2019). Primary neural degeneration in the human cochlea: evidence for hidden hearing loss in the aging ear. Neuroscience.

[18] Viana LM, O’Malley JT, Burgess BJ, Jones DD, Oliveira CA, Santos F, Merchant SN, Liberman LD, Liberman MC (2015). Cochlear neuropathy in human presbycusis: confocal analysis of hidden hearing loss in post-mortem tissue. Hear Res.

[19] Muniak MA, Ayeni FE, Ryugo DK (2018). Hidden hearing loss and endbulbs of held: evidence for central pathology before detection of ABR threshold increases. Hear Res.

[20] Salvi R, Ding D, Jiang H, Chen GD, Greco A, Manohar S, Sun W, Ralli M (2018). Hidden age-related hearing loss and hearing disorders: current knowledge and future directions. Hearing Balance Commun.

[21] Hong JW, Jeon JH, Ku CR, Noh JH, Yoo HJ, Kim DJ (2015). The prevalence and factors associated with hearing impairment in the Korean adults: the 2010-2012 Korea National Health and Nutrition Examination Survey (observational study). Medicine (Baltimore).

[22] Han C, Someya S (2013). Mouse models of age-related mitochondrial neurosensory hearing loss. Mol Cell Neurosci.

[23] Cai SQ, Sesti F (2009). Oxidation of a potassium channel causes progressive sensory function loss during aging. Nat Neurosci.

[24] Knipper M (2014). Introduction to Compensation after injury: always for good?. Neuroscience.

[25] Beisel KW, Nelson NC, Delimont DC, Fritzsch (2000) Longitudinal gradients of KCNQ4 expression in spiral ganglion and cochlear hair cells correlate with progressive hearing loss in DFNA2 10.1016/s0169-328x(00)00204-7 11042367

[26] Oliver D, Knipper M, Derst C, Fakler B (2003). Resting potential and submembrane calcium concentration of inner hair cells in the isolated mouse cochlea are set by KCNQ-type potassium channels. J Neurosci.

[27] Nouvian R, Ruel J, Wang J, Guitton MJ, Pujol R, Puel J-L (2003). Degeneration of sensory outer hair cells following pharmacological blockade of cochlear KCNQ channels in the adult guinea pig. Eur J Neurosci.

[28] Holt JR, Stauffer EA, Abraham D, Geleoc GS (2007). Dominant-negative inhibition of M-like potassium conductances in hair cells of the mouse inner ear. J Neurosci.

[29] Kharkovets T, Hardelin J-P, Safieddine S, Schweizer M, El-Amraoui A, Petit C, Jentsch TJ (2000). KCNQ4, a K+ channel mutated in a form of dominant deafness, is expressed in the inner ear and the central auditory pathway. PNAS.

[30] Kubisch C, Schroeder BC, El-Amraoui A, Marlin S, Petit C, Jentsch TJ (1999). KCNQ4, a Novel Potassium Channel Expressed in Sensory Outer Hair Cells, Is Mutated in Dominant Deafness. Cell Press.

[31] Barhanin J, Lesage F, Guillemare E, Fink M, Lazdunski M, Romey G (1996). KvLQTl and lsK (minK) proteins associate to form the IKs cardiac potassium current. Nature.

[32] Neyroud N, Tesson F, Denjoy I, Leibovici M, Donger C, Barhanin J, Fauré S, Gary F, Coumel P, Petit C, Schwartz K, Guicheney P (1997). A novel mutation in the potassium channel gene KVLQT1 causes the Jervell and Lange-Nielsen cardioauditory syndrome. Nat Genet.

[33] Sanguinetti MC, Curran ME, Zou A, Shen J, Spector PS, Atkinson DL, Keating MT (1996). Coassembly of KvLQT1 and minK {lsK} proteins to form cardiac fKs potassium channel. Nature.

[34] Maison SF, Pyott SJ, Meredith AL, Liberman MC (2013). Olivocochlear suppression of outer hair cells in vivo: evidence for combined action of BK and SK2 channels throughout the cochlea. J Neurophysiol.

[35] Wersinger E, McLean WJ, Fuchs PA, Pyott SJ (2010). BK channels mediate cholinergic inhibition of high frequency cochlear hair cells. PLoS One.

[36] Schimmang T, Duran Alonso B, Zimmermann U, Knipper M (2014). Is there a relationship between brain-derived neurotrophic factor for driving neuronal auditory circuits with onset of auditory function and the changes following cochlear injury or during aging?. Neuroscience.

[37] Macica CM, von Hehn CA, Wang L-Y, Ho C-S, Yokoyama S, Joho RH, Kaczmarek LK (2003). Modulation of the Kv3.1b potassium channel isoform adjusts the fidelity of the firing pattern of auditory neurons. J Neurosci.

[38] Dallos P (2008). Cochlear amplification, outer hair cells and prestin. Curr Opin Neurobiol.

[39] Dallos P, Zheng J, Cheatham MA (2006). Prestin and the cochlear amplifier. J Physiol.

[40] Marcotti W, Kros CJ (1999). Developmental expression of the potassium current IK,n contributes to maturation of mouse outer hair cells. J Physiol.

[41] Fang Q, Giordimaina AM, Dolan DF, Camper SA, Mustapha M (2012). Genetic background of Prop1(df) mutants provides remarkable protection against hypothyroidism-induced hearing impairment. J Assoc Res Otolaryngol.

[42] Winter H, Braig C, Zimmermann U, Geisler HS, Franzer JT, Weber T, Ley M, Engel J, Knirsch M, Bauer K, Christ S, Walsh EJ, McGee J, Kopschall I, Rohbock K, Knipper M (2006). Thyroid hormone receptors TRalpha1 and TRbeta differentially regulate gene expression of Kcnq4 and prestin during final differentiation of outer hair cells. J Cell Sci.

[43] Winter H, Braig C, Zimmermann U, Engel J, Rohbock K, Knipper M (2007). Thyroid hormone receptor alpha1 is a critical regulator for the expression of ion channels during final differentiation of outer hair cells. Histochem Cell Biol.

[44] Boettger T, Hubner CA, Maier H, Rust MB, Beck FX, Jentsch TJ (2002). Deafness and renal tubular acidosis in mice lacking the K-Cl co-transporter Kcc4. Nature.

[45] Housley GD, Ashmore JF (1992). Ionic currents of outer hair cells isolated from the guinea-pig cochlea. J Physiol.

[46] Mammano F, Ashmore JF (1996). Differential expression of outer hair cell potassium currents in the isolated cochlea of the guinea-pig. J Physiol.

[47] Kharkovets T, Dedek K, Maier H, Schweizer M, Khimich D, Nouvian R, Vardanyan V, Leuwer R, Moser T, Jentsch TJ (2006). Mice with altered KCNQ4 K+ channels implicate sensory outer hair cells in human progressive deafness. EMBO J.

[48] Gao Y, Yechikov S, Vazquez AE, Chen D, Nie L (2013). Impaired surface expression and conductance of the KCNQ4 channel lead to sensorineural hearing loss. J Cell Mol Med.

[49] Carignano C, Barila EP, Rias EI, Dionisio L, Aztiria E, Spitzmaul G (2019). Inner hair cell and neuron degeneration contribute to hearing loss in a DFNA2-like mouse model. Neuroscience.

[50] Jentsch TJ (2000). Neuronal KCNQ potassium channels - physiology and role in disease. Nat Neurosci.

[51] Ruttiger L, Sausbier M, Zimmermann U, Winter H, Braig C, Engel J, Knirsch M, Arntz C, Langer P, Hirt B, Muller M, Kopschall I, Pfister M, Munkner S, Rohbock K, Pfaff I, Rusch A, Ruth P, Knipper M (2004). Deletion of the Ca2+-activated potassium (BK) alpha-subunit but not the BKbeta1-subunit leads to progressive hearing loss. Proc Natl Acad Sci U S A.

[52] Kikuchi T, Adams JC, Miyabe Y, So E, Kobayashi T (2000). Potassium ion recycling pathway via gap junction systems in the mammalian cochlea and its interruption in hereditary nonsyndromic deafness. Med Electron Microsc.

[53] Wangemann P (2006). Supporting sensory transduction: cochlear fluid homeostasis and the endocochlear potential. J Physiol.

[54] Shen Z, Marcus DC (1998). Divalent cations inhibit IsK/KvLQT1 channels in excised membrane patches of strial marginal cells. Hear Res.

[55] Vetter DE, Mann JR, Wangemann P, Liu J, McLaughlin KJ, Lesage F, Marcus DC, Lazdunski M, Heinemann SF, Barhanin J (1996). Inner ear defects induced by null mutation of the isk gene. Neuron.

[56] Wangemann P, Liu J, Marcus DC (1995). Ion transport mechanisms responsible for K+ secretion and the transepithelial voltage across marginal cells of stria vascularis in vitro. Hear Res.

[57] Tasaki I, Spyropoulos CS (1959). Stria vascularis as source of endocochlear potential. J Neurophysiol.

[58] Nin F, Yoshida T, Sawamura S, Ogata G, Ota T, Higuchi T, Murakami S, Doi K, Kurachi Y, Hibino H (2016). The unique electrical properties in an extracellular fluid of the mammalian cochlea; their functional roles, homeostatic processes, and pathological significance. Pflugers Arch.

[59] de Castro MP, Aranega A, Franco D (2006). Protein distribution of Kcnq1, Kcnh2, and Kcne3 potassium channel subunits during mouse embryonic development. Anat Rec A Discov Mol Cell Evol Biol.

[60] Wollnik B, Schroeder BC, Kubisch C, Esperer HD, Wieacker P, Jentsch TJ (1997). Pathophysiological mechanisms of dominant and recessive KVLQT1 K+ channel mutations found in inherited cardiac arrhythmias. Hum Mol Genet.

[61] Splawski I, Tristani-Firouzi M, Lehmann MH, Sanguinetti MC, Keating MT (1997). Mutations in the hminK gene cause long QT syndrome and suppress IKs function. Nat Genet.

[62] Chouabe C, Neyroud N, Guicheney P, Lazdunski M, Romey G, Barhanin J (1997). Properties of KvLQT1 K+ channel mutations in Romano-Ward and Jervell and Lange-Nielsen inherited cardiac arrhythmias. EMBO J.

[63] Wang Q, Curran ME, Splawski I, Burn TC, Millholland JM, VanRaay TJ, Shen J, Timothy KW, Vincent GM, de Jager T, Schwartz PJ, Toubin JA, Moss AJ, Atkinson DL, Landes GM, Connors TD, Keating MT (1996). Positional cloning of a novel potassium channel gene: KVLQT1 mutations cause cardiac arrhythmias. Nat Genet.

[64] Schulze-Bahr E, Wang Q, Wedekind H, Haverkamp W, Chen Q, Sun Y, Rubie C, Hördt M, Towbin JA, Borggrefe M, Assmann G, Qu X, Somberg JC, Breithardt G, Oberti C, Funke H (1997). KCNE1 mutations cause Jervell and Lange-Nielsen syndrome. Nat Genet.

[65] Tyson J, Tranebjerg L, Bellman S, Wren C, Taylor JFN, Bathen J, Aslaksen B, Sorland SJ, Lund O, Malcolm S, Pembrey M, Bhattacharya S, BitnerGlindzicz M (1997). IsK and KVLQT1: mutation in either of the two subunits of the slow component of the delayed rectifier potassium channel can cause Jervell and Lange-Nielsen syndrome. Hum Mol Genet.

[66] Casimiro MC, Knollmann BC, Ebert SN, Vary JC, Greene AE, Franz MR, Grinberg A, Huang SP, Pfeifer K (2001). Targeted disruption of the Kcnq1 gene produces a mouse model of Jervell and Lange-Nielsen Syndrome. Proc Natl Acad Sci U S A.

[67] Wei AD, Gutman GA, Aldrich R, Chandy KG, Grissmer S, Wulff H (2005). International Union of Pharmacology. LII. Nomenclature and molecular relationships of calcium-activated potassium channels. Pharmacol Rev.

[68] Butler A, Tsunoda S, Mccobb DP, Wei A, Salkoff L (1993). Mslo, a complex mouse gene encoding maxi calcium-activated potassium channels. Science.

[69] Schumacher MA, Rivard AF, Bachinger HP, Adelman JP (2001). Structure of the gating domain of a Ca2+-activated K+ channel complexed with Ca2+/calmodulin. Nature.

[70] Dulon D, Sugasawa M, Blanchet C, Erostegui C (1995). Direct measurements of Ca(2+)-activated K+ currents in inner hair cells of the guinea-pig cochlea using photolabile Ca2+ chelators. Pflugers Arch.

[71] Wangemann P, Takeuchi S (1993). Maxi-K+ channel in single isolated cochlear efferent nerve terminals. Hear Res.

[72] Engel J, Braig C, Ruttiger L, Kuhn S, Zimmermann U, Blin N, Sausbier M, Kalbacher H, Munkner S, Rohbock K, Ruth P, Winter H, Knipper M (2006). Two classes of outer hair cells along the tonotopic axis of the cochlea. Neuroscience.

[73] Rohmann KN, Wersinger E, Braude JP, Pyott SJ, Fuchs PA (2015). Activation of BK and SK channels by efferent synapses on outer hair cells in high-frequency regions of the rodent cochlea. J Neurosci.

[74] Katz E, Elgoyhen AB, Gomez-Casati ME, Knipper M, Vetter DE, Fuchs PA, Glowatzki E (2004). Developmental regulation of nicotinic synapses on cochlear inner hair cells. J Neurosci.

[75] Hafidi A, Beurg M, Dulon D (2005). Localization and developmental expression of BK channels in mammalian cochlear hair cells. Neuroscience.

[76] Skinner LJ, Enee V, Beurg M, Jung HH, Ryan AF, Hafidi A, Dulon D (2003). Contribution of BK Ca2+-activated K+ channels to auditory neurotransmission in the guinea pig cochlea. J Neurophysiol.

[77] Kros CJ (2007). How to build an inner hair cell: challenges for regeneration. Hear Res.

[78] Marcotti W, Johnson SL, Holley MC, Kros CJ (2003). Developmental changes in the expression of potassium currents of embryonic, neonatal and mature mouse inner hair cells. J Physiol.

[79] Luneau CJ, Williams JB, Marshall J, Levitan ES, Oliva C, Smith JS, Antanavage J, Folander K, Stein RB, Swanson R (1991). Alternative splicing contributes to K+ channel diversity in the mammalian central nervous system. Proc Natl Acad Sci U S A.

[80] Gan L, Kaczmarek LK (1998). When, where, and how much? Expression of the Kv3.1 potassium channel in high-frequency firing neurons. J Neurobiol.

[81] Perney TM, Marshall J, Martin KA, Hockfield S, Kaczmarek LK (1992). Expression of the mRNAs for the Kv3.1 potassium channel gene in the adult and developing rat brain. J Neurophysiol.

[82] Weiser M, Bueno E, Sekirnjak C, Martone ME, Baker H, Hillman D, Chen S, Thornhill W, Ellisman M, Rudy B (1995). The potassium channel subunit Kv3.1b is localized to somatic and axonal membranes of specific populations of Cns neurons. J Neurosci.

[83] Ishikawa T, Nakamura Y, Saitoh N, Li WB, Iwasaki S, Takahashi T (2003). Distinct roles of Kv1 and Kv3 potassium channels at the calyx of Held presynaptic terminal. J Neurosci.

[84] Meneses D, Vega AV, Torres-Cruz FM, Barral J (2016). KV1 and KV3 potassium channels identified at presynaptic terminals of the corticostriatal synapses in rat. Neural Plast.

[85] Parameshwaran S, Carr CE, Perney TM (2001). Expression of the Kv3.1 potassium channel in the avian auditory brainstem. J Neurosci.

[86] Kanemasa T, Gan L, Perney TM, Wang LY, Kaczmarek LK (1995). Electrophysiological and pharmacological characterization of a mammalian Shaw channel expressed in NIH 3T3 fibroblasts. J Neurophysiol.

[87] Adamson CL, Reid MA, Mo ZL, Bowne-English J, Davis RL (2002). Firing features and potassium channel content of murine spiral ganglion neurons vary with cochlear location. J Comp Neurol.

[88] Flores-Otero J, Xue HZ, Davis RL (2007). Reciprocal regulation of presynaptic and postsynaptic proteins in bipolar spiral ganglion neurons by neurotrophins. J Neurosci.

[89] Friedland DR, Eernisse R, Popper P (2007). Potassium channel gene expression in the rat cochlear nucleus. Hear Res.

[90] Lu Y, Monsivais P, Tempel BL, Rubel EW (2004). Activity-dependent regulation of the potassium channel subunits Kv1.1 and Kv3.1. J Comp Neurol.

[91] Grigg JJ, Brew HM, Tempel BL (2000). Differential expression of voltage-gated potassium channel genes in auditory nuclei of the mouse brainstem. Hear Res.

[92] Liu SQ, Kaczmarek LK (1998). Depolarization selectively increases the expression of the Kv3.1 potassium channel in developing inferior colliculus neurons. J Neurosci.

[93] Elezgarai I, Díez J, Puente N, Azkue JJ, Benítez R, Bilbao A, Knöpfel T, Doñate-Oliver F, Grandes P (2003). Subcellular localization of the voltage-dependent potassium channel Kv3.1b in postnatal and adult rat medial nucleus of the trapezoid body. Neuroscience.

[94] de Villers-Sidani E, Chang EF, Bao S, Merzenich MM (2007). Critical period window for spectral tuning defined in the primary auditory cortex (A1) in the rat. J Neurosci.

[95] Hong EJ, McCord AE, Greenberg ME (2008). A biological function for the neuronal activity-dependent component of Bdnf transcription in the development of cortical inhibition. Neuron.

[96] Lehmann K, Steinecke A, Bolz J (2012). GABA through the ages: regulation of cortical function and plasticity by inhibitory interneurons. Neural Plast.

[97] Xu H, Kotak VC, Sanes DH (2010). Normal hearing is required for the emergence of long-lasting inhibitory potentiation in cortex. J Neurosci.

[98] Griffen TC, Maffei A (2014). GABAergic synapses: their plasticity and role in sensory cortex. Front Cell Neurosci.

[99] Knipper M, van Dijk P, Schulze H, Mazurek B, Krauss P, Scheper V, Warnecke A, Schlee W, Schwabe K, Singer W, Braun C, Delano PH, Fallgatter AJ, Ehlis AC, Searchfield GD, Munk MHJ, Baguley DM, Ruttiger L (2020). The neural bases of tinnitus: lessons from deafness and cochlear implants. J Neurosci.

[100] Massengill JL, Smith MA, Son DI, ODowd DK (1997). Differential expression of K4-AP currents and Kv3.1 potassium channel transcripts in cortical neurons that develop distinct firing phenotypes. J Neurosci.

[101] Rudy B, McBain CJ (2001). Kv3 channels: voltage-gated K+ channels designed for high-frequency repetitive firing. Trends Neurosci.

[102] McBain CJ, Fisahn A (2001). Interneurons unbound. Nat Rev Neurosci.

[103] Brew HM, Forsythe ID (1995). Two voltage-dependent K+ conductances with complementary functions in postsynaptic integration at a central auditory synapse. J Neurosci.

[104] Perney TM, Kaczmarek LK (1997). Localization of a high threshold potassium channel in the rat cochlear nucleus. J Comp Neurol.

[105] Wang LY, Gan L, Forsythe ID, Kaczmarek LK (1998). Contribution of the Kv3.1 potassium channel to high-frequency firing in mouse auditory neurones. J Physiol.

[106] von Hehn CA, Bhattacharjee A, Kaczmarek LK (2004). Loss of Kv3.1 tonotopicity and alterations in cAMP response element-binding protein signaling in central auditory neurons of hearing impaired mice. J Neurosci.

[107] Keithley EM (2020). Pathology and mechanisms of cochlear aging. J Neurosci Res.

[108] Turner JG, Parrish JL, Hughes LF, Toth LA, Caspary DM (2005). Hearing in laboratory animals: strain differences and nonauditory effects of noise. Comp Med.

[109] Boettcher FA, Mills JH, Norton BL (1993). Age-related changes in auditory evoked potentials of gerbils. I. Response amplitudes. Hear Res.

[110] Boettcher FA, Mills JH, Norton BL, Schmiedt RA (1993). Age-related changes in auditory evoked potentials of gerbils. II. Response latencies. Hear Res.

[111] Heeringa AN, Koppl C (2019). The aging cochlea: towards unraveling the functional contributions of strial dysfunction and synaptopathy. Hear Res.

[112] Gleich O, Semmler P, Strutz J (2016). Behavioral auditory thresholds and loss of ribbon synapses at inner hair cells in aged gerbils. Exp Gerontol.

[113] Mohrle D, Ni K, Varakina K, Bing D, Lee SC, Zimmermann U, Knipper M, Ruttiger L (2016). Loss of auditory sensitivity from inner hair cell synaptopathy can be centrally compensated in the young but not old brain. Neurobiol Aging.

[114] Ruttiger L, Panford-Walsh R, Schimmang T, Tan J, Zimmermann U, Rohbock K, Kopschall I, Limberger A, Muller M, Fraenzer JT, Cimerman J, Knipper M (2007). BDNF mRNA expression and protein localization are changed in age-related hearing loss. Neurobiol Aging.

[115] Wu T, Marcus DC (2003). Age-related changes in cochlear endolymphatic potassium and potential in CD-1 and CBA/CaJ mice. J Assoc Res Otolaryngol.

[116] Kujawa SG, Liberman MC (2006). Acceleration of age-related hearing loss by early noise exposure: evidence of a misspent youth. J Neurosci.

[117] Wang Y, Ren C (2012). Effects of repeated “benign” noise exposures in young CBA mice: shedding light on age-related hearing loss. J Assoc Res Otolaryngol.

[118] Yoshida N, Hequembourg SJ, Atencio CA, Rosowski JJ, Liberman MC (2000). Acoustic injury in mice: 129/SvEv is exceptionally resistant to noise-induced hearing loss. Hear Res.

[119] Benkafadar N, Francois F, Affortit C, Casas F, Ceccato JC, Menardo J, Venail F, Malfroy-Camine B, Puel JL, Wang J (2019). ROS-induced activation of DNA damage responses drives senescence-like state in postmitotic cochlear cells: implication for hearing preservation. Mol Neurobiol.

[120] Henderson D, Bielefeld EC, Harris KC, Hu BH (2006). The role of oxidative stress in noise-induced hearing loss. Ear Hear.

[121] Vlajkovic SM, Lin SC, Wong AC, Wackrow B, Thorne PR (2013). Noise-induced changes in expression levels of NADPH oxidases in the cochlea. Hear Res.

[122] Roth TN (2015). Aging of the auditory system. Handb Clin Neurol.

[123] Han BR, Lin SC, Espinosa K, Thorne PR, Vlajkovic SM (2019) Inhibition of the adenosine A2A receptor mitigates excitotoxic injury in organotypic tissue cultures of the rat cochlea. Cells 8. 10.3390/cells8080877 10.3390/cells8080877 PMC6721830 31408967

[124] Bures Z, Grecova J, Popelar J, Syka J (2010). Noise exposure during early development impairs the processing of sound intensity in adult rats. Eur J Neurosci.

[125] Grecova J, Bures Z, Popelar J, Suta D, Syka J (2009). Brief exposure of juvenile rats to noise impairs the development of the response properties of inferior colliculus neurons. Eur J Neurosci.

[126] Marchetta P, Mohrle D, Eckert P, Reimann K, Wolter S, Tolone A, Lang I, Wolters M, Feil R, Engel J, Paquet-Durand F, Kuhn M, Knipper M, Ruttiger L (2020). Guanylyl cyclase A/cGMP signaling slows hidden, age- and acoustic trauma-induced hearing loss. Front Aging Neurosci.

[127] Liberman LD, Liberman MC (2019). Cochlear efferent innervation is sparse in humans and decreases with age. J Neurosci.

[128] Maison SF, Luebke AE, Liberman MC, Zuo J (2002). Efferent protection from acoustic injury is mediated via α9 nicotinic acetylcholine receptors on outer hair cells. J Neurosci.

[129] Maison SF, Liberman MC (2000). Predicting vulnerability to acoustic injury with a noninvasive assay of olivocochlear reflex strength. J Neurosci.

[130] Vergara C, Latorre R, Marrion NV, Adelman JP (1998). Calcium-activated potassium channels. Curr Opin Neurobiol.

[131] Typlt M, Mirkowski M, Azzopardi E, Ruettiger L, Ruth P, Schmid S (2013). Mice with deficient BK channel function show impaired prepulse inhibition and spatial learning, but normal working and spatial reference memory. PLoS One.

[132] Knipper M, Panford-Walsh R, Singer W, Ruttiger L, Zimmermann U (2015). Specific synaptopathies diversify brain responses and hearing disorders: you lose the gain from early life. Cell Tissue Res.

[133] Robertson D (2009). Centrifugal control in mammalian hearing. Clin Exp Pharmacol Physiol.

[134] Suga N, Gao E, Zhang Y, Ma X, Olsen JF (2000). The corticofugal system for hearing: recent progress. Proc Natl Acad Sci U S A.

[135] Kong JH, Adelman JP, Fuchs PA (2008). Expression of the SK2 calcium-activated potassium channel is required for cholinergic function in mouse cochlear hair cells. J Physiol.

[136] Jeng JY, Johnson SL, Carlton AJ, De Tomasi L, Goodyear RJ, De Faveri F, Furness DN, Wells S, Brown SDM, Holley MC, Richardson GP, Mustapha M, Bowl MR, Marcotti W (2020) Age-related changes in the biophysical and morphological characteristics of mouse cochlear outer hair cells. J Physiol. 10.1113/JP279795 10.1113/JP279795 PMC7612122 32608086

[137] Nakazawa K, Spicer SS, Schulte BA (1995). Ultrastructural localization of Na,K-ATPase in the gerbil cochlea. J Histochem Cytochem.

[138] Spicer SS, Schulte BA (2002). Spiral ligament pathology in quiet-aged gerbils. Hear Res.

[139] Ding B, Walton JP, Zhu X, Frisina RD (2018). Age-related changes in Na, K-ATPase expression, subunit isoform selection and assembly in the stria vascularis lateral wall of mouse cochlea. Hear Res.

[140] Ryan AF, Watts AG (1991). Expression of mRNAs encoding alpha and beta subunit isoforms of the Na,K-ATPase in the rat cochlea. Mol Cell Neurosci.

[141] Gratton MA, Smyth BJ, Lam CF, Boettcher FA, Schmiedt RA (1997). Decline in the endocochlear potential corresponds to decreased Na,K-ATPase activity in the lateral wall of quiet-aged gerbils. Hear Res.

[142] Gratton MA, Smyth BJ, Schulte BA, Vincent DA (1995). Na,K-ATPase activity decreases in the cochlear lateral wall of quiet-aged gerbils. Hear Res.

[143] Prazma J, Carrasco VN, Butler B, Waters G, Anderson T, Pillsbury HC (1990). Cochlear microcirculation in young and old gerbils. Arch Otolaryngol Head Neck Surg.

[144] Yang H, Xiong H, Huang Q, Pang J, Zheng X, Chen L, Yu R, Zheng Y (2013). Compromised potassium recycling in the cochlea contributes to conservation of endocochlear potential in a mouse model of age-related hearing loss. Neurosci Lett.

[145] Knipper M, Claussen C, Ruttiger L, Zimmermann U, Lullmann-Rauch R, Eskelinen EL, Schroder J, Schwake M, Saftig P (2006). Deafness in LIMP2-deficient mice due to early loss of the potassium channel KCNQ1/KCNE1 in marginal cells of the stria vascularis. J Physiol.

[146] Zettel ML, Zhu X, O’Neill WE, Frisina RD (2007). Age-related decline in Kv3.1b expression in the mouse auditory brainstem correlates with functional deficits in the medial olivocochlear efferent system. J Assoc Res Otolaryngol.

[147] Spongr VP, Flood DG, Frisina RD, Salvi RJ (1997). Quantitative measures of hair cell loss in CBA and C57BL/6 mice throughout their life spans. J Acoust Soc Am.

[148] Jung DK, Lee SY, Kim D, Joo KM, Cha CI, Yang HS, Lee WB, Chung YH (2005). Age-related changes in the distribution of Kv1.1 and Kv3.1 in rat cochlear nuclei. Neurol Res.

[149] Costa M, Lepore F, Prevost F, Guillemot JP (2016). Effects of aging on peripheral and central auditory processing in rats. Eur J Neurosci.

[150] Hu H, Gan J, Jonas P (2014). Interneurons. Fast-spiking, parvalbumin(+) GABAergic interneurons: from cellular design to microcircuit function. Science.

[151] Martin del Campo HN, Measor KR, Razak KA (2012). Parvalbumin immunoreactivity in the auditory cortex of a mouse model of presbycusis. Hear Res.

[152] Strain GM, McGee KA (2017). Distortion product otoacoustic emissions in young adult and geriatric cats. Vet J.

[153] Gonzalez C, Baez-Nieto D, Valencia I, Oyarzun I, Rojas P, Naranjo D, Latorre R (2012). K(+) channels: function-structural overview. Compr Physiol.

[154] Van Eyken E, Van Laer L, Fransen E, Topsakal V, Lemkens N, Laureys W, Nelissen N, Vandevelde A, Wienker T, Van De Heyning P, Van Camp G (2006). KCNQ4: a gene for age-related hearing impairment?. Hum Mutat.

[155] Beisel KW, Rocha-Sanchez SM, Morris KA, Nie L, Feng F, Kachar B, Yamoah EN, Fritzsch B (2005). Differential expression of KCNQ4 in inner hair cells and sensory neurons is the basis of progressive high-frequency hearing loss. J Neurosci.

[156] Van Laer L, Carlsson PI, Ottschytsch N, Bondeson ML, Konings A, Vandevelde A, Dieltjens N, Fransen E, Snyders D, Borg E, Raes A, Van Camp G (2006). The contribution of genes involved in potassium-recycling in the inner ear to noise-induced hearing loss. Hum Mutat.

[157] Walter S, Atzmon G, Demerath EW, Garcia ME, Kaplan RC, Kumari M, Lunetta KL, Milaneschi Y, Tanaka T, Tranah GJ, Volker U, Yu L, Arnold A, Benjamin EJ, Biffar R, Buchman AS, Boerwinkle E, Couper D, De Jager PL, Evans DA, Harris TB, Hoffmann W, Hofman A, Karasik D, Kiel DP, Kocher T, Kuningas M, Launer LJ, Lohman KK, Lutsey PL, Mackenbach J, Marciante K, Psaty BM, Reiman EM, Rotter JI, Seshadri S, Shardell MD, Smith AV, van Duijn C, Walston J, Zillikens MC, Bandinelli S, Baumeister SE, Bennett DA, Ferrucci L, Gudnason V, Kivimaki M, Liu Y, Murabito JM, Newman AB, Tiemeier H, Franceschini N (2011). A genome-wide association study of aging. Neurobiol Aging.

[158] Alders M, Bikker H, Christiaans I (2003) Long QT Syndrome. GeneReviews® [Internet]. University of Washington, Seattle, Seattle (WA) 20301308

[159] Lee MP, Ravenel JD, Hu RJ, Lustig LR, Tomaselli G, Berger RD, Brandenburg SA, Litzi TJ, Bunton TE, Limb C, Francis H, Gorelikow M, Gu H, Washington K, Argani P, Goldenring JR, Coffey RJ, Feinberg AP (2000). Targeted disruption of the Kvlqt1 gene causes deafness and gastric hyperplasia in mice. J Clin Invest.

[160] Ullrich S, Su J, Ranta F, Wittekindt OH, Ris F, Rosler M, Gerlach U, Heitzmann D, Warth R, Lang F (2005). Effects of I(Ks) channel inhibitors in insulin-secreting INS-1 cells. Pflugers Arch.

[161] Hanson RL, Guo T, Muller YL, Fleming J, Knowler WC, Kobes S, Bogardus C, Baier LJ (2013). Strong parent-of-origin effects in the association of KCNQ1 variants with type 2 diabetes in American Indians. Diabetes.

[162] Li YY, Wang XM, Lu XZ (2014). KCNQ1 rs2237892 C-->T gene polymorphism and type 2 diabetes mellitus in the Asian population: a meta-analysis of 15,736 patients. J Cell Mol Med.

[163] Unoki H, Takahashi A, Kawaguchi T, Hara K, Horikoshi M, Andersen G, Ng DP, Holmkvist J, Borch-Johnsen K, Jorgensen T, Sandbaek A, Lauritzen T, Hansen T, Nurbaya S, Tsunoda T, Kubo M, Babazono T, Hirose H, Hayashi M, Iwamoto Y, Kashiwagi A, Kaku K, Kawamori R, Tai ES, Pedersen O, Kamatani N, Kadowaki T, Kikkawa R, Nakamura Y, Maeda S (2008). SNPs in KCNQ1 are associated with susceptibility to type 2 diabetes in East Asian and European populations. Nat Genet.

[164] Voight BF, Scott LJ, Steinthorsdottir V, Morris AP, Dina C, Welch RP, Zeggini E, Huth C, Aulchenko YS, Thorleifsson G, McCulloch LJ, Ferreira T, Grallert H, Amin N, Wu G, Willer CJ, Raychaudhuri S, McCarroll SA, Langenberg C, Hofmann OM, Dupuis J, Qi L, Segre AV, van Hoek M, Navarro P, Ardlie K, Balkau B, Benediktsson R, Bennett AJ, Blagieva R, Boerwinkle E, Bonnycastle LL, Bengtsson Bostrom K, Bravenboer B, Bumpstead S, Burtt NP, Charpentier G, Chines PS, Cornelis M, Couper DJ, Crawford G, Doney AS, Elliott KS, Elliott AL, Erdos MR, Fox CS, Franklin CS, Ganser M, Gieger C, Grarup N, Green T, Griffin S, Groves CJ, Guiducci C, Hadjadj S, Hassanali N, Herder C, Isomaa B, Jackson AU, Johnson PR, Jorgensen T, Kao WH, Klopp N, Kong A, Kraft P, Kuusisto J, Lauritzen T, Li M, Lieverse A, Lindgren CM, Lyssenko V, Marre M, Meitinger T, Midthjell K, Morken MA, Narisu N, Nilsson P, Owen KR, Payne F, Perry JR, Petersen AK, Platou C, Proenca C, Prokopenko I, Rathmann W, Rayner NW, Robertson NR, Rocheleau G, Roden M, Sampson MJ, Saxena R, Shields BM, Shrader P, Sigurdsson G, Sparso T, Strassburger K, Stringham HM, Sun Q, Swift AJ, Thorand B, Tichet J, Tuomi T, van Dam RM, van Haeften TW, van Herpt T, van Vliet-Ostaptchouk JV, Walters GB, Weedon MN, Wijmenga C, Witteman J, Bergman RN, Cauchi S, Collins FS, Gloyn AL, Gyllensten U, Hansen T, Hide WA, Hitman GA, Hofman A, Hunter DJ, Hveem K, Laakso M, Mohlke KL, Morris AD, Palmer CN, Pramstaller PP, Rudan I, Sijbrands E, Stein LD, Tuomilehto J, Uitterlinden A, Walker M, Wareham NJ, Watanabe RM, Abecasis GR, Boehm BO, Campbell H, Daly MJ, Hattersley AT, Hu FB, Meigs JB, Pankow JS, Pedersen O, Wichmann HE, Barroso I, Florez JC, Frayling TM, Groop L, Sladek R, Thorsteinsdottir U, Wilson JF, Illig T, Froguel P, van Duijn CM, Stefansson K, Altshuler D, Boehnke M, McCarthy MI, investigators M, Consortium G (2010). Twelve type 2 diabetes susceptibility loci identified through large-scale association analysis. Nat Genet.

[165] Kong A, Steinthorsdottir V, Masson G, Thorleifsson G, Sulem P, Besenbacher S, Jonasdottir A, Sigurdsson A, Kristinsson KT, Jonasdottir A, Frigge ML, Gylfason A, Olason PI, Gudjonsson SA, Sverrisson S, Stacey SN, Sigurgeirsson B, Benediktsdottir KR, Sigurdsson H, Jonsson T, Benediktsson R, Olafsson JH, Johannsson OT, Hreidarsson AB, Sigurdsson G, Consortium D, Ferguson-Smith AC, Gudbjartsson DF, Thorsteinsdottir U, Stefansson K (2009). Parental origin of sequence variants associated with complex diseases. Nature.

[166] Provenzano MJ, Domann FE (2007). A role for epigenetics in hearing: establishment and maintenance of auditory specific gene expression patterns. Hear Res.

[167] Hermann A, Sitdikova GF, Weiger TM (2015). Oxidative stress and maxi calcium-activated potassium (BK) channels. Biomolecules.

[168] Honrath B, Matschke L, Meyer T, Magerhans L, Perocchi F, Ganjam GK, Zischka H, Krasel C, Gerding A, Bakker BM, Bunemann M, Strack S, Decher N, Culmsee C, Dolga AM (2017). SK2 channels regulate mitochondrial respiration and mitochondrial Ca(2+) uptake. Cell Death Differ.

[169] Rizzuto R, De Stefani D, Raffaello A, Mammucari C (2012). Mitochondria as sensors and regulators of calcium signalling. Nat Rev Mol Cell Biol.

[170] Lemasters JJ, Theruvath TP, Zhong Z, Nieminen AL (2009). Mitochondrial calcium and the permeability transition in cell death. Biochim Biophys Acta.

[171] Muona M, Berkovic SF, Dibbens LM, Oliver KL, Maljevic S, Bayly MA, Joensuu T, Canafoglia L, Franceschetti S, Michelucci R, Markkinen S, Heron SE, Hildebrand MS, Andermann E, Andermann F, Gambardella A, Tinuper P, Licchetta L, Scheffer IE, Criscuolo C, Filla A, Ferlazzo E, Ahmad J, Ahmad A, Baykan B, Said E, Topcu M, Riguzzi P, King MD, Ozkara C, Andrade DM, Engelsen BA, Crespel A, Lindenau M, Lohmann E, Saletti V, Massano J, Privitera M, Espay AJ, Kauffmann B, Duchowny M, Moller RS, Straussberg R, Afawi Z, Ben-Zeev B, Samocha KE, Daly MJ, Petrou S, Lerche H, Palotie A, Lehesjoki AE (2015). A recurrent de novo mutation in KCNC1 causes progressive myoclonus epilepsy. Nat Genet.

[172] Poirier K, Viot G, Lombardi L, Jauny C, Billuart P, Bienvenu T (2017). Loss of Function of KCNC1 is associated with intellectual disability without seizures. Eur J Hum Genet.

[173] Karlsson KK, Harris JR, Svartengren M (1997). Description and primary results from an audiometric study of male twins. Ear Hear.

[174] Vuckovic D, Mezzavilla M, Cocca M, Morgan A, Brumat M, Catamo E, Concas MP, Biino G, Franze A, Ambrosetti U, Pirastu M, Gasparini P, Girotto G (2018). Whole-genome sequencing reveals new insights into age-related hearing loss: cumulative effects, pleiotropy and the role of selection. Eur J Hum Genet.

[175] Wells HRR, Freidin MB, Zainul Abidin FN, Payton A, Dawes P, Munro KJ, Morton CC, Moore DR, Dawson SJ, Williams FMK (2019). GWAS identifies 44 independent associated genomic loci for self-reported adult hearing difficulty in UK Biobank. Am J Hum Genet.

[176] Serrano F, Klann E (2004). Reactive oxygen species and synaptic plasticity in the aging hippocampus. Ageing Res Rev.

[177] Matalon S, Hardimann KM, Jain L, Eaton DC, Kotlikoff M, Eu JP, Sun J, Meissner G, Stamler JS (2003). Regulation of ion channel structure and function by reactive oxygen-nitrogen species. Am J Phys Lung Cell Mol Phys.

[178] Barrese V, Stott JB, Greenwood IA (2018). KCNQ-encoded potassium channels as therapeutic targets. Annu Rev Pharmacol Toxicol.

[179] Greene DL, Hoshi N (2017). Modulation of Kv7 channels and excitability in the brain. Cell Mol Life Sci.

[180] Shin DH, Jung J, Koh YI, Rim JH, Lee JS, Choi HJ, Joo SY, Yu S, Cha DH, Lee SY, Lee JH, Lee MG, Choi JY, Gee HY (2019). A recurrent mutation in KCNQ4 in Korean families with nonsyndromic hearing loss and rescue of the channel activity by KCNQ activators. Hum Mutat.

[181] Xia X, Zhang Q, Jia Y, Shu Y, Yang J, Yang H, Yan Z (2020). Molecular basis and restoration of function deficiencies of Kv7.4 variants associated with inherited hearing loss. Hear Res.

[182] Lange W, Geissendorfer J, Schenzer A, Grotzinger J, Seebohm G, Friedrich T, Schwake M (2009). Refinement of the binding site and mode of action of the anticonvulsant Retigabine on KCNQ K+ channels. Mol Pharmacol.

[183] Schenzer A, Friedrich T, Pusch M, Saftig P, Jentsch TJ, Grotzinger J, Schwake M (2005). Molecular determinants of KCNQ (Kv7) K+ channel sensitivity to the anticonvulsant retigabine. J Neurosci.

[184] Wuttke TV, Seebohm G, Bail S, Maljevic S, Lerche H (2005). The new anticonvulsant retigabine favors voltage-dependent opening of the Kv7.2 (KCNQ2) channel by binding to its activation gate. Mol Pharmacol.

[185] Leitner MG, Feuer A, Ebers O, Schreiber DN, Halaszovich CR, Oliver D (2012). Restoration of ion channel function in deafness-causing KCNQ4 mutants by synthetic channel openers. Br J Pharmacol.

[186] Cooke PS, Nanjappa MK, Ko C, Prins GS, Hess RA (2017). Estrogens in male physiology. Physiol Rev.

[187] Rapetti-Mauss R, O’Mahony F, Sepulveda FV, Urbach V, Harvey BJ (2013). Oestrogen promotes KCNQ1 potassium channel endocytosis and postendocytic trafficking in colonic epithelium. J Physiol.

[188] Curhan SG, Eliassen AH, Eavey RD, Wang M, Lin BM, Curhan GC (2017). Menopause and postmenopausal hormone therapy and risk of hearing loss. Menopause.

[189] Li Y, Chen B, Zou W, Wang X, Wu Y, Zhao D, Sun Y, Liu Y, Chen L, Miao L, Yang C, Wang X (2016). The lysosomal membrane protein SCAV-3 maintains lysosome integrity and adult longevity. J Cell Biol.

[190] Patel R, McKinnon BJ (2018). Hearing loss in the elderly. Clin Geriatr Med.

[191] Ambrosi G, Ghezzi C, Zangaglia R, Levandis G, Pacchetti C, Blandini F (2015). Ambroxol-induced rescue of defective glucocerebrosidase is associated with increased LIMP-2 and saposin C levels in GBA1 mutant Parkinson’s disease cells. Neurobiol Dis.

[192] Zeng X-H, Xia X-M, Lingle CJ (2003). Redox-sensitive extracellular gates formed by auxiliary β subunits of calcium-activated potassium channels. Nat Struct Biol.

[193] Lingle CJ, Martinez-Espinosa PL, Yang-Hood A, Boero LE, Payne S, Persic D, Babak VG, Xiao M, Zhou Y, Xia XM, Pyott SJ, Rutherford MA (2019). LRRC52 regulates BK channel function and localization in mouse cochlear inner hair cells. Proc Natl Acad Sci U S A.

[194] Blank T, Nijholt I, Kye MJ, Radulovic J, Spiess J (2003). Small-conductance, Ca2+-activated K+ channel SK3 generates age-related memory and LTP deficits. Nat Neurosci.

[195] Zhang WH, Liu WZ, He Y, You WJ, Zhang JY, Xu H, Tian XL, Li BM, Mei L, Holmes A, Pan BX (2019). Chronic stress causes projection-specific adaptation of amygdala neurons via small-conductance calcium-activated potassium channel downregulation. Biol Psychiatry.

[196] Coles B, Wilton LA, Good M, Chapman PF, Wann KT (2008). Potassium channels in hippocampal neurones are absent in a transgenic but not in a chemical model of Alzheimer’s disease. Brain Res.

[197] Chambers AR, Pilati N, Balaram P, Large CH, Kaczmarek LK, Polley DB (2017). Pharmacological modulation of Kv3.1 mitigates auditory midbrain temporal processing deficits following auditory nerve damage. Sci Rep.

[198] Parthasarathy A, Cunningham PA, Bartlett EL (2010). Age-related differences in auditory processing as assessed by amplitude-modulation following responses in quiet and in noise. Front Aging Neurosci.

[199] Abbott GW, Mercer EAJ, Miller RT, Ramesh B, Srai SKS (1998). Conformational changes in a mammalian voltage-dependent potassium channel inactivation peptide. Biochemistry.

[200] Duprat F, Guillemare E, Romey G, Fink M, Lesage F, Lazdunski M (1995). usceptibilityofclonedK+ channelstoreactiveoxygenspecies. Proc Natl Acad Sci USA.

[201] Pan Y, Weng J, Cao Y, Bhosle RC, Zhou M (2008). Functional coupling between the Kv1.1 channel and aldoketoreductase Kvbeta1. J Biol Chem.

[202] Medrihan L, Umschweif G, Sinha A, Reed S, Lee J, Gindinova K, Sinha SC, Greengard P, Sagi Y (2020). Reduced Kv3.1 activity in dentate gyrus parvalbumin cells induces vulnerability to depression. Biol Psychiatry.

[203] Kann O (2016). The interneuron energy hypothesis: Implications for brain disease. Neurobiol Dis.

[204] Boddum K, Hougaard C, Xiao-Ying Lin J, von Schoubye NL, Jensen HS, Grunnet M, Jespersen T (2017). Kv3.1/Kv3.2 channel positive modulators enable faster activating kinetics and increase firing frequency in fast-spiking GABAergic interneurons. Neuropharmacology.

[205] Brown MR, El-Hassar L, Zhang Y, Alvaro G, Large CH, Kaczmarek LK (2016). Physiological modulators of Kv3.1 channels adjust firing patterns of auditory brain stem neurons. J Neurophysiol.

[206] Dubno JR, Eckert MA, Lee FS, Matthews LJ, Schmiedt RA (2013). Classifying human audiometric phenotypes of age-related hearing loss from animal models. J Assoc Res Otolaryngol.

